# Endothelium-dependent Effect of Sesame Seed Feeding on Vascular Reactivity of Streptozotocin-diabetic Rats: Underlying Mechanisms 

**Published:** 2013

**Authors:** Mehrdad Roghani, Mohammad Reza Jalali-Nadoushan, Tourandokht Baluchnejadmojarad, Mohammad-Reza Vaez Mahdavi, Gholamali Naderi, Farshad Roghani Dehkordi, Mohammad Taghi Joghataei

**Affiliations:** a ***Department of Physiology, School of Medicine and Medicinal Plant Research Center, Shahed University, Tehran, Iran.***; b ***Department of Pathology, School of Medicine, Shahed University, Tehran, Iran.***; c ***Department of Physiology, School of Medicine, Tehran University of Medical Sciences, Tehran, Iran. ***; d ***Department of Biochemistry, School of Medicine, Shahed University, Tehran, Iran.***; e ***Department of Cardiology, School of Medicine, Isfahan University of Medical Sciences, Tehran, Iran. ***; f ***Department of Anatomy and Neuroscience, Cellular and Molecular Research Center, School of Medicine, Tehran University of Medical Sciences, Tehran, Iran. ***

**Keywords:** *Sesamum indicum L*, Sesame seed, Diabetes mellitus, Streptozotocin, Aorta

## Abstract

Cardiovascular disorders continue to constitute major causes of morbidity and mortality in diabetic patients. In this study, the effect of chronic administration of sesame (*Sesamum indicum L*) seed feeding was studied on aortic reactivity of streptozotocin (STZ)-diabetic rats. Male diabetic rats received sesame seed-mixed food at weight ratios of 3% and 6% for 7 weeks, one week after diabetes induction. Contractile responses to KCl and phenylephrine (PE) and relaxation response to acetylcholine (ACh) and sodium nitroprusside (SNP) were obtained from aortic rings. Maximum contractile response of endothelium-intact rings to PE was significantly lower in sesame-treated diabetic rats (at a ratio of 6%) relative to untreated diabetics and endothelium removal abolished this difference. Endothelium-dependent relaxation to ACh was also significantly higher in sesame-treated diabetic rats (at a ratio of 6%) as compared to diabetic rats and pretreatment of rings with nitric oxide synthase inhibitor, N(G)-nitro-l-arginine methyl ester (L-NAME) significantly attenuated the observed response. Two-month diabetes also resulted in an elevation of malondialdehyde (MDA) and decreased superoxide dismutase (SOD) activity and sesame treatment significantly reversed the increased MDA content and restored activity of SOD. We thus conclude that chronic treatment of diabetic rats with sesame seed could in a dose-manner prevent some abnormal changes in vascular reactivity through nitric oxide and via attenuation of oxidative stress in aortic tissue and endothelium integrity is necessary for this beneficial effect.

## Introduction

Cardiovascular disorders continue to constitute major causes of morbidity and mortality in diabetic patients in spite of significant achievements in their diagnosis and treatment (1). Changes in vascular responsiveness to vasoconstrictors and vasodilators are mainly responsible for the development of some vascular complications of diabetics (2). Most of these complications are due to increased serum glucose and augmented generation of reactive oxygen species (ROS), that finally lead to endothelial dysfunction (3). 

Sesame (*Sesamum indicum *L.) is a flowering plant of the genus *Sesamum *and is one of the oldest cultivated plants in the world that is mainly grown for its oil rich edible seeds. The seeds are widely used as spice. Sesame seed is the oldest condiment known to man and is probably the first crop grown for its edible oil (4). The seeds possess potent antioxidant effects due to the presence of active constituents (within the group lignans) such as sesamin and sesamol, which are the phytoestrogens with antioxidant and anti-cancer properties (5). Sesame seeds also contain phytosterols that reduce blood cholesterol level (6). The oil is said to be laxative and promotes menstruation (6). Sesamin, one of the major lignans in sesame seed and oil and its isomers have beneficial physiological effects, acting as antioxidants (7), anti-carcinogens (8), anti-hypertensives (9, 10) and are capable of reducing serum lipids (11). It has also indicated that sesame fractions could enhance plasma levels of *α*- and *γ*-tocopherol in rats (12). Recent works demonstrated that sesame metabolites induce a nitric oxide-dependent vasorelaxation in an *in vitro *system (13) and some sesame constituents such as sesamin could enhance endothelium-dependent relaxation in deoxycorticosterone acetate (DOCA)-salt hypertensive rats (10). It has also been reported that the aqueous extract of leaves from sesame induces dose-dependent vasorelaxation in guinea-pig aorta (14). Nevertheless, the exact underlying mechanisms of an *in-vivo *protective effect of sesame seed on vascular system are not understood. Therefore, this study was designed to assess, for the first time, the beneficial effect of chronic sesame seed feeding on the improvement of aortic reactivity of STZ-diabetic rats and to investigate some underlying mechanisms.

## Experimental

*Animals*

Male albino Wistar rats (n= 48) (Pasteur’s institute, Tehran, IR Iran), weighing 235-300 g, were housed in an air-conditioned colony room at 21 ± 2 °C and supplied with standard pellet diet and tap water *ad libitum*. Procedures involving animals and their care were conducted in conformity with NIH guidelines for the care and use of laboratory animals.

*Experimental protocol*

The rats were rendered diabetic by a single intraperitoneal dose of 60 mg kg^-1^ STZ freshly dissolved in ice-cold 0.1 M citrate buffer (pH 4.5). Age-matched normal animals that received an injection of an equivalent volume of buffer comprised a non-diabetic control group. One week after STZ injection, overnight fasting blood samples were collected and serum glucose concentration was measured using glucose oxidation method (Zistchimie, Tehran). Only those animals with a serum glucose level higher than 250 mg/dL were considered as diabetic. During the subsequent weeks, diabetes was reconfirmed by the presence of polyphagia, polydipsia, polyuria, and weight loss. Normal and hyperglycemic rats (a total of 48) were randomly allocated and similarly grouped into six groups (eight in each): normal vehicle-treated control, sesame-treated controls in two subgroups, diabetic, and sesame-treated diabetics in two subgroups. Sesame seed powder was mixed with standard food at weight ratios of 3% and 6% and food was freely available to rats throughout the experimental period for 7 weeks. Changes in body weight were regularly recorded during the study. The rats were finally anesthetized with diethyl ether, decapitated, and through opening the abdomen, descending thoracic aorta was carefully excised and placed in a petri dish filled with cold Krebs solution containing (in mM): NaCl 118.5, KCl 4.7, CaCl_2_ 1.5, MgSO_4_ 1.2, KH_2_PO_4_ 1.2, NaHCO_3_ 25, and glucose 11. The aorta was cleaned of excess connective tissue and fat and cut into rings of approximately 4 mm in length. Aortic rings were suspended between the bases of two triangular-shaped wires. One wire was attached to a fixed tissue support in a 50 mL isolated tissue bath containing Krebs solution (pH 7.4) maintained at 37 °C and continuously aerated with a mixture of 5% CO_2_ and 95% O_2_. The other end of each wire was knotted to a cotton thread to a F60 isometric force transducer (Narco Biosystems, USA) connected to a computer. In all experiments, special care was taken to avoid damage to the luminal surface of endothelium. Aortic rings were equilibrated at a resting tension of 1.5 g for at least 45 min. In some experiments, the endothelium was mechanically removed by gently rubbing the internal surface with a filter paper. Isometric contractions were induced by the addition of phenylephrine (PE, 1 μM) and once the contraction was stabilized, a single concentration of acetylcholine (1 μM) was added to the bath in order to assess the endothelial integrity of the preparations. Endothelium was considered to be intact when this drug elicited a vasorelaxation ≥ 50% of the maximal contraction obtained in vascular rings precontracted with PE. The absence of acetylcholine relaxant action in the vessels indicated the total removal of endothelial cells. After assessing the integrity of the endothelium, vascular tissues were allowed to recuperate for at least 30 min. 

At the end of the equilibration period, dose–response curves for KCl (10-50 mM) and PE (10^-10^-10^-5^ M) in the presence and absence of endothelium were obtained in aortic rings in a cumulative manner. To evaluate ACh- (10^-9^-10^-4^ M) and SNP- (10^-9^-10^-4^ M) induced vasodilatation in rings with and without endothelium, they were preconstricted with a submaximal concentration of PE (10^-6^ M) which produced 70-80% of maximal response. The sensitivity to the agonists was evaluated as pD2, which is the negative logarithm of the concentration of the drug required to produce 50% of the maximum response.

To determine the participation of NO, rings were incubated for 30 min before the experiment with L-NAME (100 μM, a non-selective NOS inhibitor). To determine the participation of endothelial vasodilator factors in response to ACh, aortic segments were incubated with INDO (10 μM, an inhibitor of COX-derived prostanoid synthesis) 30 min before exposure to ACh.

After each vasoreactivity experiment, aortic rings were blotted, weighed, and the cross-sectional area (csa) was calculated using the following formula: Cross-sectional area (mm^2^) = weight (mg) × [length (mm) × density (mg mm^3^-1)]^-1^. The density of the preparations was 1.05 mg/mm^2^.

*Determination of MDA concentration in aortic rings*

After removing aortic segments and cleaning them of extra tissues, they were blotted dry and weighed, then made into 5% tissue homogenate in ice-cold 0.9% saline solution. A supernatant was obtained from tissue homogenate by centrifugation (1000×g, 4 ºC, 5 min). The MDA concentration (thiobarbituric acid reactive substances, TBARS) in the supernatant was measured as described before (15). Briefly, trichloroacetic acid and TBARS reagent were added to supernatant, then mixed and incubated at 100 ºC for 80 min. After cooling on ice, samples were centrifuged at 1000×g for 20 min and the absorbance of the supernatant was read at 532 nm. TBARS results were expressed as MDA equivalents using tetraethoxypropane as standard. 

*Measurement of SOD activity in aortic rings*

The supernatant of tissue homogenate were obtained as described earlier (16). Briefly, supernatant was incubated with xanthine and xanthine oxidase in potassium phosphate buffer (pH 7.8, 37 ºC) for 40 min and NBT was added. Blue formazan was then monitored spectrophotometrically at 550 nm. The amount of protein that inhibited NBT reduction to 50% maximum was defined as 1 nitrite unit (NU) of SOD activity.

*Drugs*

Phenylephrine, streptozotocin, ACh, INDO, and L-NAME were purchased from Sigma Chemical (St. Louis, Mo., USA). All other chemicals were purchased from Merck (Germany) and Darupakhsh Co. (Iran). Indomethacin solution was prepared in ethanol in such a way that the maximal ethanol concentration of the medium was less than 0.001% (v/v). 

*Data and statistical analysis*

All values were given as means ± SEM. Contractile response to PE was expressed as grams of tension per cross-sectional area of tissue. Relaxation response for ACh was expressed as a percentage decrease of the maximum contractile response induced by PE. Statistical analysis was carried out using repeated measure ANOVA and one-way ANOVA followed by Tukey post-hoc test. A statistical p value less than 0.05 considered significant.

## Results

**Figure 1:**
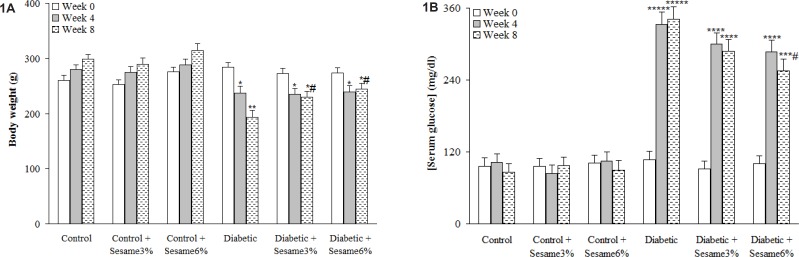
Body weight and serum glucose concentration at different weeks (mean ± SEM). * p < 0.05, ** p < 0.005, *** p < 0.001, **** p < 0.0005, ***** p < 0.00001 (as compared to week 0 in the same group) # p < 0.05 (Versus diabetic in the same week).

After 8 weeks, the weight of the vehicle-treated diabetic rats was found to be significantly lower as compared to control group (p < 0.005) and sesame treatment at both weight ratios, especially at a ratio of 6%, caused a significant reduction in diabetic rats as compared to vehicle-treated diabetics (p < 0.05). Untreated diabetic rats had also an elevated serum glucose level over those of control rats (p < 0.0001) and treatment of diabetic rats with sesame at a weight ratio of 6% caused a significant decrease in the serum glucose relative to diabetics only at week 8 (p < 0.05). In addition, sesame treatment of control rats did not produce any significant change in serum glucose level (Figure 1). 

**Figure 2:**
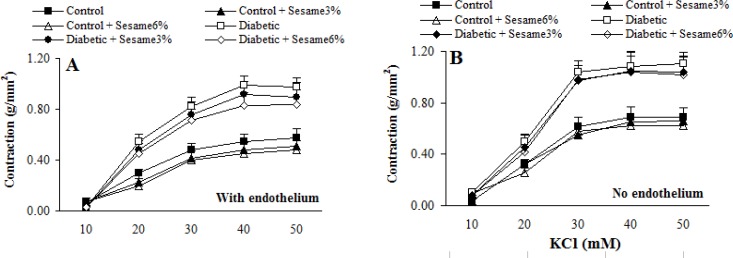
Cumulative concentration-response curves for KCl in aortic preparations 8 weeks after experiment in the presence (A) and absence (B) of endothelium (mean ± SEM).

**Figure 3:**
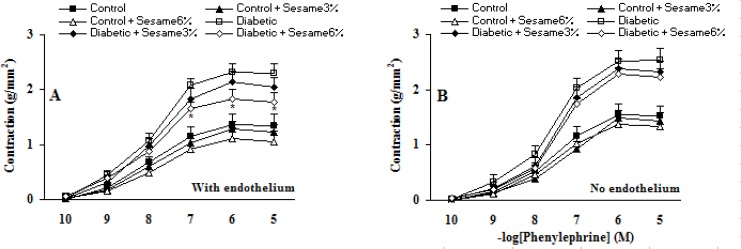
Cumulative concentration-response curves for PE in aortic preparations 8 weeks after experiment in the presence (A) and absence (B) of endothelium (mean ± SEM). * p < 0.05 (as compared to diabetic).

Cumulative addition of KCl (10-50 mM) and PE (10^-10^-10^-5^ M) resulted in concentration dependent contractions in aortas of all groups (Figures 2-3). The maximum contractile responses to KCl and PE in the aortas from vehicle-treated diabetic rats in the presence of endothelium were found to be significantly (p < 0.01-0.005) greater than vehicle-treated control rats and concentration-response curve of endothelium-intact aortas from sesame-treated diabetic rats (at a ratio of 6%) to PE (and not to KCl) was significantly attenuated compared to vehicle-treated diabetics (p < 0.05). Although endothelium-denuded aortic rings in all groups showed a higher contractile response to KCl and PE, however, the observed changes between treated and untreated diabetics were attenuated after endothelium removal. This clearly indicated the necessity of endothelium presence for beneficial vascular effect of sesame seed. In addition, aortic rings with endothelium from sesame-treated control group showed a non-significant reduction in contractile response to KCl and PE as compared to vehicle-treated controls. There were also no significant differences among the groups in terms of the pD2 (data not shown), indicating that there has not been any significant change in the sensitivity of aortic rings from different groups.

**Figure 4:**
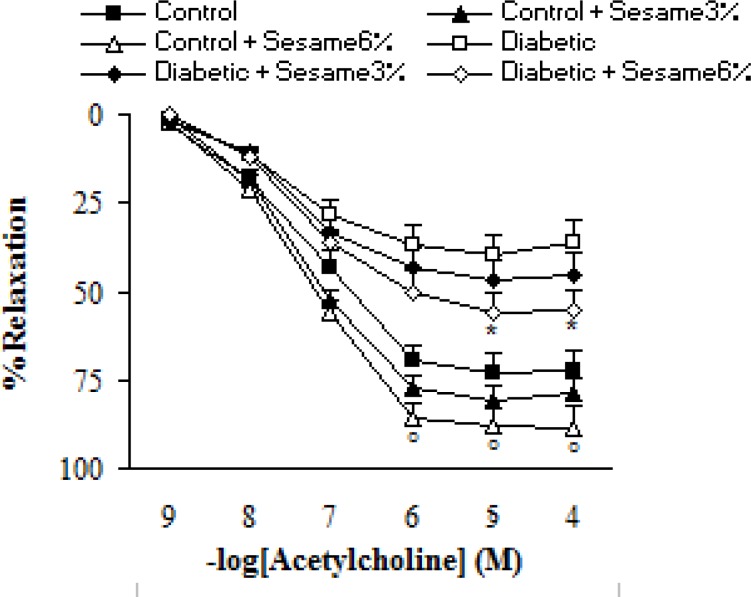
Cumulative concentration-response curves for ACh in endothelium-intact aortic rings precontracted with PE 8 weeks after experiment. Relaxation responses are expressed as a percentage of the submaximal contraction induced by phenylephrine which produced 70-80% of maximal response (mean ± SEM). * p < 0.05 (as compared to diabetic).

Addition of ACh resulted in concentration-dependent relaxations in all aortic rings precontracted with PE (Figure 4). As was expected, endothelium-dependent relaxation response induced by ACh was significantly lower in vehicle-treated diabetic rats compared to the vehicle-treated controls (p < 0.05-0.005). Meanwhile, the existing difference between sesame-treated (at a weight ratio of 6%) and vehicle-treated diabetic rats was only significant (p < 0.05) at concentrations higher than 10^-5^ M. Meanwhile, relaxation response of sesame-treated control rats was non-significantly greater than the control group. 

**Figure 5:**
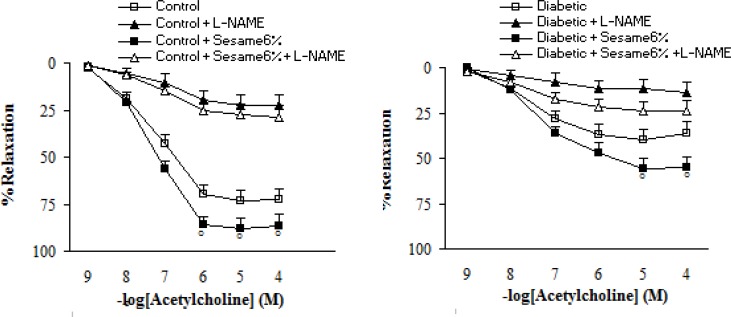
Cumulative concentration-response curves for ACh in endothelium-intact aortic rings precontracted with phenylephrine in the presence and absence of L-NAME 8 weeks after the experiment in control and diabetic rats. Relaxation responses are expressed as a percentage of the submaximal contraction induced by phenylephrine which produced 70-80% of maximal response (mean ± SEM). * p < 0.05 (as compared to diabetic).

Pre-incubation of aortic rings with L-NAME non-significantly increased the contractile response of aortic rings from all groups to PE. However, this increase was non-significantly lower in sesame-treated diabetic group (at a weight ratio of 6%) as compared to the vehicle-treated diabetics (data not shown). Regarding the relaxation response to ACh, pre-incubation of aortic rings with L-NAME almost completely abolished the vasodilator response to ACh in segments from sesame-treated diabetic rats (at a weight ratio of 6%), indicating the important role of endothelium-derived NO in the vascular effect of sesame seed (Figure 5).

**Figure 6:**
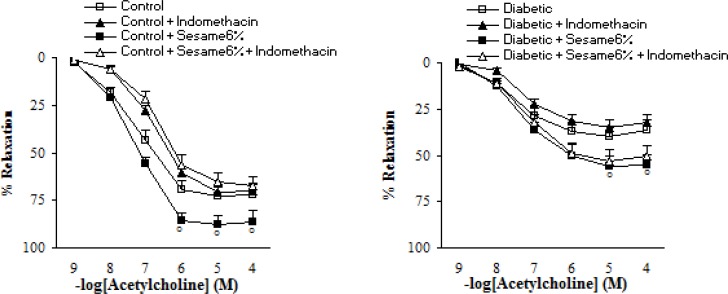
Maximum relaxation response to acetylcholine (ACh) in aortic rings precontracted with phenylephrine in the presence and absence of indomethacin (INDO) eight weeks after experiment in control and diabetic rats. Relaxation responses are expressed as a percentage (mean ± SEM). * p < 0.05 (as compared to diabetic).

 Pre-incubation of aortic segments from sesame-treated diabetic rats (at a weight ratio of 6%) with INDO partly and non-significantly diminished the endothelial vasodilator response to ACh (Figure 6). 

Regarding aortic lipid peroxidation markers (Table 1), STZ-induced diabetes resulted in an elevation of MDA content and decreased SOD activity (p < 0.005-0.001) in aortic tissue and chronic treatment of diabetic group with sesame seed at a weight ratio of 6% significantly reversed the increased MDA content and restored activity of SOD (p < 0.05).

**Table 1:** Malondialdehyde (MDA) content and superoxide dismutase (SOD) activity in aortic tissue of studied groups

**Groups**	**MDA (μmol.g** ^-1^ **protein)**	**SOD activity (kNU.g** ^-1^ **protein)**
Control (n=7)	5.7 ± 0.5	117 ± 6
Control + Sesame6% (n=5)	5.3 ± 0.6	123 ± 8
Diabetic (n=6)	9.2 ± 0.7^**^	76 ± 8^*^
Diabetic + Sesame6% (n=7)	6.7 ± 0.6^#^	108 ± 7^#^

* p < 0.005, ** p < 0.001 (vs. control group); # p < 0.05 (vs. diabetic group)

## Discussion

In this study, administration of sesame for 7 weeks did have a weak and significant hypoglycemic effect, it reduced the enhanced contractility of aortic rings to PE and increased ACh-induced relaxation, which was partly due to involvement of NO pathway, since the relaxation was blocked in the presence of L-NAME. In the presence of indomethacin, relaxation response to ACh was non-significant and partly attenuated. In addition, endothelium removal clearly affected KCl- and PE-induced contractions in sesame-treated diabetic rats. Regarding oxidative stress markers, sesame treatment at a weight ratio of 6% attenuated the increased MDA content and restored activity of SOD.

Vascular dysfunction is one of the complicating features of diabetes in humans and its experimental model and hyperglycemia is the primary cause of micro and macrovascular complications in diabetic condition (17). Compared to the aortic rings from control animals, contraction of aortas to KCl and PE from diabetic rats significantly increased, that was consistent with previous studies (15) and chronic sesame administration was capable to attenuate this change only for PE-induced contractions. Impaired endothelial function (18), enhanced sensitivity of calcium channels (19), an increase in vasoconstrictor prostanoids due to increased superoxide anions and increased sensitivity to adrenergic agonists (20) might all be responsible for increased contractile responses in diabetic rats, which could have been improved following sesame treatment. 

In endothelial cells of most of the vascular beds, ACh can stimulate production and release of endothelial-derived relaxing factors including nitric oxide (NO), prostacyclin and endothelium-derived hyperpolarizing factor and in this way leads to relaxation of vascular smooth muscle in an endothelium-dependent manner (21-23). The ACh-induced relaxation response is endothelium-dependent and NO-mediated (15). The results of this work revealed that the endothelium-dependent relaxant response was reduced in aortas from STZ-induced diabetic rats and this reduced relaxation was partially recovered by sesame treatment. Although some researchers have asserted that the sensitivity to acetylcholine decreases in diabetes (20), the results of this research, in accordance with those of many previous ones (24) reveals that diabetic condition in long-term only decreases the maximum responses to ACh but not the sensitivity (pD2). 

Impaired endothelium-dependent relaxation in STZ-induced diabetic rat might be due to increased blood glucose level and decreased blood insulin level. It has been shown that hyperglycaemia causes tissue damage by several mechanisms, including advanced glycation end product (AGE) formation, increased polyol pathway flux, apoptosis and reactive oxygen species (ROS) formation (25). Our results showed that sesame treatment could exert a significantly weak hypoglycemic effect in STZ-induced diabetic rats. Therefore, its beneficial effect on aortic tissue of diabetic rats should be in part due to its hypoglycemic effect. Some damaging effect of diabetes on vascular tissue of diabetic animals is also believed to be due to enhanced oxidative stress, as shown by enhanced MDA and decreased activity of defensive enzymes such as SOD (16), as was observed in this study. This could also lead to diabetes-induced functional changes in vascular endothelial cells and the development of altered endothelium-dependent vasoreactivity. The results of the present study also showed that chronic treatment with sesame at a ratio of 6% significantly decreased MDA content and enhanced SOD activity in aortic tissue from diabetic rats, indicating that the improvement in vascular responsiveness from sesame may be partly due to amelioration of lipid peroxidation and oxidative injury. These results clearly suggested another mechanism for the effect of sesame on the improvement of endothelial dysfunction which could be related to its antioxidant activity.

In conclusion, to the best of our knowledge, this is the first study to report *in-vivo *chronic treatment of diabetic rats with sesame dose- and endothelium-dependent could prevent the functional changes in vascular reactivity observed in diabetic rats through nitric oxide- and not prostaglandin-dependent pathways and via attenuation of aortic lipid peroxidation. Our data may be helpful in the development of new natural drugs from sesame to improve endothelial function and to prevent cardiovascular diseases.
